# Efficacy and toxicity of immune checkpoint inhibitors combination therapy for advanced renal cell carcinoma: a systematic review and network meta-analysis

**DOI:** 10.3389/fimmu.2024.1255577

**Published:** 2024-02-08

**Authors:** Xiangyu Chen, Zhunan Xu, Changgui Wu, Lijun Xie, Pengyu Wang, Xiaoqiang Liu

**Affiliations:** Department of Urology, Tianjin Medical University General Hospital, Tianjin, China

**Keywords:** immune checkpoint inhibitors, tyrosine kinase inhibitors, advanced renal cell carcinoma, combination therapy, efficacy

## Abstract

**Background:**

Although immune checkpoint inhibitors (ICIs) show a significant overall survival advantage over standard advanced renal cell carcinoma (aRCC) therapies, tumor response to these agents remains poor. Some studies have shown that combination therapy including an ICI appears to be the best treatment; however, the overall benefit in terms of efficacy and toxicity still needs to be assessed. Thus, we performed a network meta-analysis to evaluate the differences in the efficacy of several combinations that include an ICI to provide a basis for clinical treatment selection.

**Methods:**

We conducted a thorough search of PubMed, EMBASE, and the Cochrane Library for articles from January 2010 to June 2023. R 4.4.2 and STATA 16.0 were used to analyze data; hazard ratio (HR) and odds ratio (OR) with 95% confidence intervals (CI) were used to assess the results.

**Results:**

An indirect comparison showed that nivolumab plus cabozantinib and pembrolizumab plus lenvatinib were the most effective treatments for progression-free survival (PFS), with no significant differences between the two interventions (HR, 1.31; 95% CI, 0.96–1.78; P=0.08); rank probability showed that pembrolizumab plus lenvatinib had a 57.1% chance of being the preferred treatment. In the absence of indirect comparisons between pembrolizumab plus axitinib, nivolumab plus ipilimumab, avelumab plus axitinib, nivolumab plus cabozantinib, and pembrolizumab plus lenvatinib, pembrolizumab plus axitinib (40.2%) was the best treatment option for overall survival (OS). Compared to pembrolizumab plus lenvatinib, nivolumab plus ipilimumab (OR, 0.07; 95% CI, 0.01–0.65; P=0.02) and pembrolizumab plus axitinib (OR, 0.05; 95% CI, 0.00–0.78; P<0.001) had a lower incidence of overall adverse events (AEs).

**Conclusion:**

Pembrolizumab plus lenvatinib and pembrolizumab plus axitinib resulted in the highest PFS and OS rates, respectively. Pembrolizumab plus axitinib may be the best option when AEs are a concern.

**Systematic review registration:**

https://inplasy.com/, identifier INPLASY202410078.

## Introduction

Kidney cancer is among the 10 most common cancers in both men and women, representing 4.2% of all new cancer cases. It is estimated that approximately 81800 people will be diagnosed with kidney cancer by 2023 in the United States (1). Renal cell carcinoma (RCC) is the most common form of kidney cancer, accounting for 90% of all tumors (2). Nearly 35% of patients present metastatic disease at diagnosis, and as many as 40% develop metastasis after primary surgical treatment of localized RCC (3). Although prognosis for patients with advanced RCC (aRCC) has improved significantly over the past decade, the vast majority of patients will ultimately die from their disease. Thus, there is an urgent need to investigate additional treatment options (4, 5).

With the introduction and regulatory approval of drugs that target the vascular endothelial growth factor (VEGF) or mammalian target of rapamycin (mTOR) pathways and considerably increase the objective response rate (ORR) and/or median progression-free survival (PFS) compared to earlier therapeutic modalities, the treatment of aRCC has advanced significantly (6, 7). Standard of care therapies now include orally available multitargeted tyrosine kinase inhibitors (TKIs) such as sunitinib, pazopanib, axitinib, and cabozantinib, and the mTOR inhibitors everolimus and temsirolimus (8). However, these therapies are limited by innate and acquired resistance, which usually occurs during the first year of treatment, and durable and complete responses (CRs) to these targeted therapies are rare (9). Given the immune responsiveness of RCC, immune checkpoint inhibitors (ICIs) such as anti-programmed death receptor 1 (PD-1), anti-programmed death receptor ligand 1 (PD-L1), and anti-cytotoxic T lymphocyte antigen 4 (CTLA-4) are highly promising therapeutic options. Nivolumab, the first ICI to be approved by the U.S. Food and Drug Administration (FDA) and the European Commission, showed superior overall survival (OS) to everolimus in a phase III clinical study (NCT01668784, CheckMate 025); median OS was 25 months (95% CI 21.8–NR) with nivolumab versus 19.6 months (95% CI 17.6–23.1) with everolimus (10). Despite the positive Checkmate 025 results, only 1% of nivolumab-treated patients experienced CRs, and only 31% of patients experienced durable responses lasting longer than 12 months. Owing to the complex and dynamic nature of the tumor immune response, the use of combination therapy including an ICI to enhance antitumor effects appears to be feasible (11). However, the overall effectiveness of ICI-based combination therapies needs to be assessed. To provide a basis for clinical treatment options for patients with aRCC, we conducted an indirect comparison and network meta-analysis to evaluate the efficacy and toxicity of combination therapies with different ICIs.

## Methods

### Definition of the outcome

The objective of this analysis was to assess whether ICI-based combination therapy resulted in better outcomes than sunitinib alone in patients with aRCC. For each trial, the combination therapy was considered the experimental arm and sunitinib was the control. OS and PFS were considered the primary endpoints according to the Response Evaluation Criteria in Solid Tumors (RECIST), version 1.1 (12). The key secondary endpoints were ORR and adverse events (AEs). In addition, we conducted a subgroup analysis according to age and sex to highlight any differences in survival outcomes between ICI-based combinations and sunitinib. Our systematic review is registered at https://inplasy.com/(registration number is INPLASY202410078; DOI number is 10.37766/inplasy2024.1.0078).

### Selection of the studies

**Figure 1:**
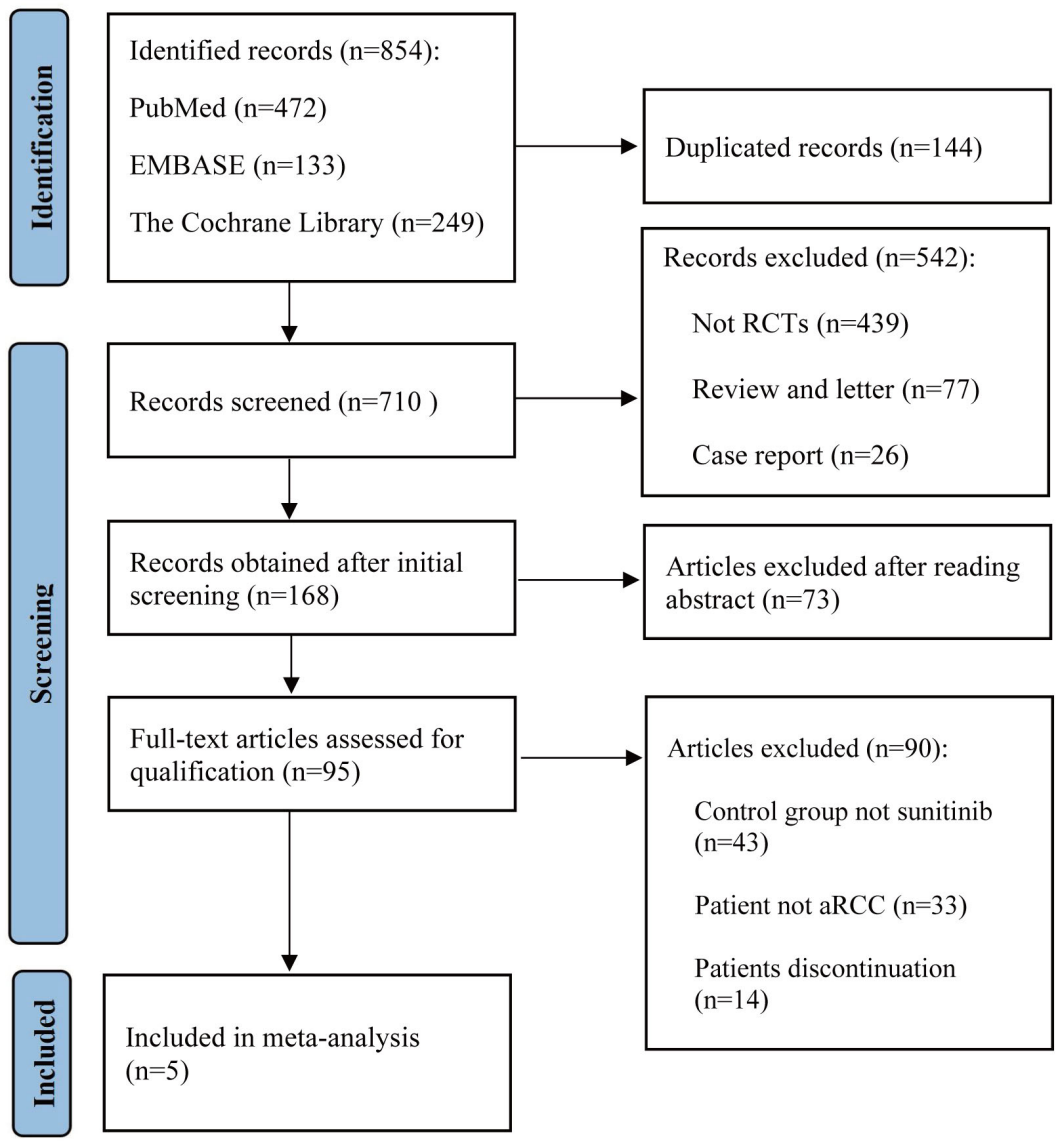
Flow chart for PRISMA-based screening of articles.

We conducted a thorough search of PubMed, EMBASE, and the Cochrane Library for articles from January 2020 up to June 2023, using a combination of the following keywords: immune checkpoint inhibitors, renal cell carcinoma, sunitinib, pembrolizumab, nivolumab, avelumab, and ipilimumab. Detailed search strategies were listed inside the **Supplementary Material**. A list of article titles and abstracts evaluated for relevance in light of the given research selection criteria was produced. The entire texts of the screened papers were assessed for inclusion in the study. **Figure 1** illustrates the search process following the PRISMA guidelines. For studies published in different journals with overlapping data, duplicate data, or the same authors, we used the most recent and comprehensive study.

### Inclusion and exclusion criteria

The inclusion criteria for eligible studies were as follows: (a) randomized controlled design; (b) inclusion of only aRCC patients; (c) provision of at least one of the following oncologic outcomes: PFS or OS; (d) inclusion of both primary and secondary endpoints; and (e) hazard ratios (HR) or event counts can be extracted from the study. The exclusion criteria were as follows: (a) insufficient primary data or incomplete data in the study; (b) publications containing duplicated or low-quality information; (c) studies not including ICI-based combination therapies, and (d) reviews, letters, commentaries, or case reports.

### Data extraction and study quality

**Figure 2:**
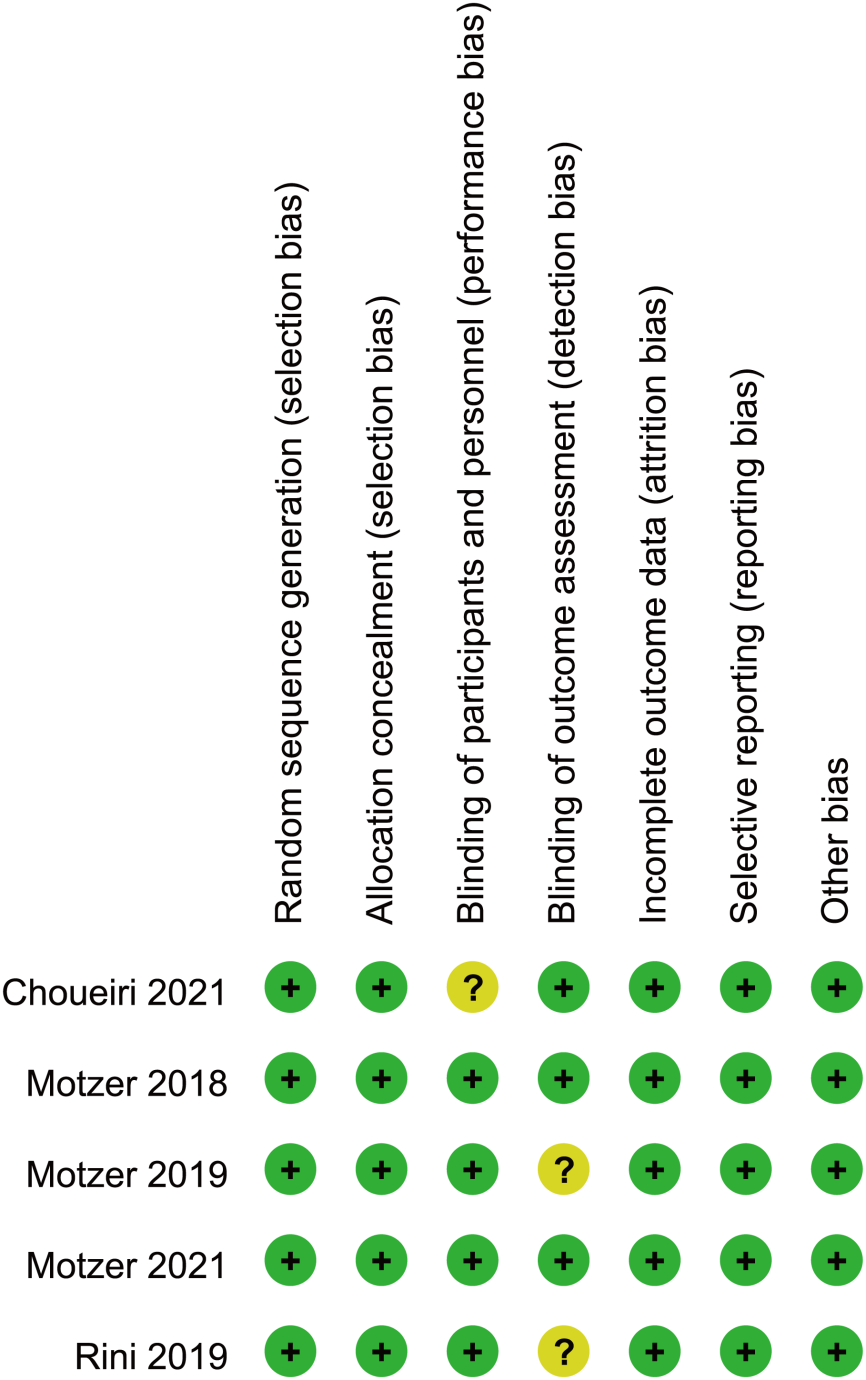
Bias risk assessment criteria for randomized controlled trials based on the Cochrane Collaborative Network.

Two researchers independently screened the literature and extracted data according to the PRISMA statement. The reasons for excluding the articles were also recorded. Disagreements were resolved by consulting a third-party expert. The following information was extracted from each article: name of the first author, year of publication, name of the clinical trial, median year of patients, intervention and control arms, sarcomatoid features, and International Metastatic Renal Cell Carcinoma Database Consortium (IMDC) prognostic risk. For each study, HRand confidence intervals (CI) of the primary endpoints were extracted, including OS and PFS, whereas for ORR, the number of patients who experienced complete response, partial response, and AEs was extracted. The quality of the included trials was assessed using the Cochrane Collaboration tool to assess the risk of bias in randomized controlled trials (**Figure 2**) (13).

### Statistical analysis

Stata 16.0 and R 4.4.2 were used to analyze the data. HR, odds ratio (OR), and 95% CI were used as measures of effect size for all included studies. For indirect comparison of selected endpoints, we performed a Bayesian network meta-analysis using the GeMTC package in R. Considering that there was only one data point per intervention, no source of heterogeneity was assessed; therefore, indirect comparisons were made uniformly using a fixed-effects model. If there were no differences in indirect comparisons, rank probability (for OS and PFS) or surface under the cumulative ranking (SUCRA) (for ORR, complete response, partial response, and AEs) was used to provide a posterior probability of each intervention for selected outcomes.

## Results

### Study characteristics

A total of 854 articles were identified following a search of the databases. After removing duplicates and adding articles found in the reference lists, we evaluated articles by full-text review, and five articles were included in the analysis (14–18). A total of 3957 study participants were assigned to receive pembrolizumab plus axitinib, nivolumab plus ipilimumab, avelumab plus axitinib, nivolumab plus cabozantinib, pembrolizumab plus lenvatinib, or sunitinib. The characteristics of these studies are summarized in **Table 1**. Eligible patients were aged 18 years or older, had newly diagnosed or recurrent aRCC, and had not received any previous systemic therapy for advanced disease. **Table 2** summarizes the results for each study endpoint. **Table 3** summarizes the indirect comparison results of different interventions at each endpoint. **Table 4** summarizes the probability rankings of the interventions.

**Table 1:** Characteristics of included trials in the meta-analysis.

First author	Year	Clinical trial	Median age—yr	Intervention arm	Control arm	Tumor PD-L1 expression(%)	Previous nephrectomy—%
Rini	2019	KEYNOTE-426	62	Pembrolizumab-Axitinib	Sunitinib	≥1(59.3) <1(40.7)	82.6
Motzer	2018	CheckMate 214	62	Nivolumab-Ipilimumab	Sunitinib	≥1(26) <1(74)	80
Motzer	2019	NCT02684006	62	Avelumab-Axitinib	Sunitinib	—	79.6
Choueiri	2021	CheckMate 9ER	62	Nivolumab-Cabozantinib	Sunitinib	≥1(25.7) <1 or indeterminate(74.3)	68.7
Motzer	2021	CLEAR	64	Pembrolizumab-Lenvatinib	Sunitinib	≥1(30.1) <1(31.5) Not available(38.3)	73.8

**Table 2:** Summary of each endpoint included in the trial.

	KEYNOTE-426				^h^CheckMate 214				NCT02684006				CheckMate 9ER				CLEAR			
	^a^P-A(n=432)	^f^S(n=429)	HR(95% CI)	P	^b^N-I(n=425)	^f^S(n=422)	HR(95% CI)	P	^c^Ave-A(n=442)	^f^S(n=444)	HR(95% CI)	P	^d^N-C(n=323)	^f^S(n=328)	HR(95% CI)	P	^e^P-L(n=355)	^f^S(n=357)	HR(95% CI)	P
Overall survival (mo)	^Rg^NR	NR	0.53(0.38-0.74)	<0.0001	NR	26.0	0.63(0.44-0.89)	<0.001	—	—	—	—	NR	NR	0.60(0.40-0.89)	0.001	NR	NR	0.66(0.49-0.88)	0.005
Progression-free survival (mo)	15.1	11.1	0.69(0.57-0.84)	<0.001	11.6	8.4	0.82(0.64-1.05)	0.03	13.8	8.4	0.69(0.56-0.84)	<0.001	16.6	8.3	0.51(0.41-0.64)	<0.001	23.9	9.2	0.39(0.32-0.49)	<0.001
Objrctive response rate (%)	59.3	35.7	—	—	42.0	27.0	—	—	51.5	25.7	—	—	55.7	27.1	—	—	71.0	36.1	—	—
Complete response (n)	25	8	—	—	40	107	—	—	15	8	—	—	26	15	—	—	57	15	—	—
Partial response (n)	231	145	—	—	137	317/320	—	—	212	106	—	—	154	74	—	—	195	114	—	—
Overall adverse events (n)	422/429	423/425	—	—	509/547	521/535	—	—	432/434	436/439	—	—	319/320	317/320	—	—	351/352	335/340	—	—
Adverse events ≥3 (n)	325/429	325/429	—	—	250/547	335/535	—	—	309/434	314/439	—	—	241/320	226/320	—	—	290/352	244/340	—	—

a P-A, Pembrolizumab-Axitinib; ^b^N-I, Nivolumab-Ipilimumab; ^c^Ave-A, Avelumab-Axitinib; ^d^N-C, Nivolumab-Cabozantinib; ^e^P-L, Pembrolizumab-Lenvatinib; ^f^S, Sunitinib.

g NR, Not reached.

h In CheckMate 214 trial, some endpoints (overall survival; progression-free survival; objrctive response rate; complete response; partial response) included data from intermediate and poor-risk patients; adverse events were for all intention-to-treat population.

**Table 3:** Results of indirect comparisons of five interventions at each endpoint.

	P-A vs. N-I	P-A vs. Ave-A	P-A vs N-C	P-A vs P-L	N-I vs Ave-A	N-I vs N-C	N-I vs P-L	Ave-A vs N-C	Ave-A vs P-L	N-C vs P-L
HR (95% CI)										
**Overall survival**	0.84(0.52-1.37)	—	0.88(0.52-1.49)	0.80(0.52-1.25)	—	1.05(0.62-1.79)	0.95(0.60-1.51)	—	—	0.91(0.55-1.49)
<65 years	0.89(0.52-1.51)	—	1.07(0.58-1.97)	—	—	1.20(0.73-2.00)	—	—	—	—
≥65 years	0.67(0.36-1.24)	—	0.66(0.33-1.30)	—	—	0.98(0.54-1.78)	—	—	—	—
Male	0.76(0.48-1.21)	—	0.92(0.53-1.57)	—	—	1.20(0.76-1.90)	—	—	—	—
Female	0.87(0.42-1.80)	—	0.66(0.29-1.50)	—	—	0.76(0.38-1.53)	—	—	—	—
**Progression-free survival**	0.84(0.61-1.15)	1.00(0.76-1.32)	1.35(1.01-1.82)	1.77(1.33-2.36)	1.19(0.86-1.64)	1.61(1.15-2.24)	2.10(1.52-2.91)	1.35(1.00-1.83)	1.77(1.32-2.37)	1.31(0.96-1.78)
<65 years	—	1.17(0.78-1.74)	1.59(1.09-2.33)	1.89(1.30-2.76)	—	—	—	1.36 (0.90- 2.07)	1.62 (1.07- 2.45)	1.19 (0.80- 1.77)
≥65 years	—	0.89(0.51-1.53)	0.93(0.57-1.51)	1.47(0.91-2.36)	—	—	—	1.04 (0.60- 1.83)	1.65 (0.95- 2.86)	1.58 (0.97- 2.59)
Male	—	1.38(0.95-1.99)	1.60(1.13-2.27)	2.03(1.45-2.84)	—	—	—	1.17 (0.79- 1.72)	1.47 (1.01- 2.15)	1.26 (0.88- 1.80)
female	—	0.60(0.32-1.12)	0.89(0.38-1.58)	29 (0.71- 2.33)	—	—	—	1.48 (0.77- 2.83)	2.14 (1.10- 4.16)	1.45 (0.78- 2.70)
OR (95% CI)										
**Objective response rate**	1.33(0.90-1.99)	0.86(0.58-1.27)	0.78(0.51-1.19)	0.61(0.40-0.92)	0.64(0.43-0.96)	0.58(0.38-0.90)	0.45(0.30-0.70)	0.90(0.59-1.39)	0.71(0.46-1.08)	0.78(0.50-1.23)
**Complete response**	0.37(0.11-1.29)	1.69(0.52-5.53)	1.77(0.63-5.01)	0.74(0.27-2.01)	4.53(1.26-16.27)	4.74(1.51-14.92)	1.99(0.66-6.03)	1.05(0.35-3.11)	0.44(0.15-1(25)	0.42(0.17-1.01)
**Partial response**	1.61(1.07-2.41)	0.77(0.51-1.14)	0.72(0.47-1.11)	0.87(0.57-1.31)	0.48(0.31-0.72)	0.45(0.28-0.70)	0.54(0.35-0.83)	0.94(0.60-1.46)	1.13(0.74-1.72)	1.20(0.76-1.90)
**Overall adverse events**	0.79(0.15-4.32)	0.19(0.02-2.09)	0.09(0.01-1.50)	0.05(0.00-0.78)	0.24(0.04-1.62)	0.12(0.01-1.25)	0.07(0.01-0.65)	0.49(0.03-8.88)	0.28(0.02-4.67)	0.58(0.03-13.14)
**Adverse events ≥3**	2.59(1.76-3.82)	1.32(0.87-2.02)	1.03(0.65.1.63)	0.71(0.44-1.13)	0.51(0.35-0.75)	0.40(0.26-0.61)	0.27(0.18-0.42)	0.78(0.49-1.22)	0.53(0.34-0.85)	0.69(0.42-1.14)

P-A, Pembrolizumab-Axitinib; N-I, Nivolumab-Ipilimumab; Avelumab-Axitinib(Ave-A); N-C, Nivolumab-Cabozantinib; P-L, Pembrolizumab-Lenvatinib.

**Table 4:** Rank probability or Surface Under the Cumulative Ranking (SUCRA) values for interventions.

	P-A	N-I	Ave-A	N-C	P-L	S	Inference
^a^Overall survival (%)	40.2	19.7	—	25.3	14.9	0.0	P-A>N-C>N-I>P-L>S
^a^Progression-free survival (%)	8.4	5.3	8.1	21.1	57.1	0.0	P-L>N-C>P-A=Ave-A>N-I>S
^b^Objrctive response rate (%)	45.3	22.0	62.7	74.1	95.8	0.0	P-L>N-C>Ave-A>P-A>N-I>S
^b^Complete response (%)	60.2	96.1	34.5	32.2	75.0	2.1	N-I>P-L>P-A>Ave-A=N-C>S
^b^Partial response (%)	47.8	20.0	80.5	86.7	64.7	0.3	N-C=Ave-A>P-L>P-A>N-I>S
^b^Overall adverse events (%)	87.8	85.5	39.9	23.9	13.1	49.9	P-A=N-I>S>Ave-A>N-C>P-L
^b^Adverse events ≥3 (%)	30.5	100.0	66.1	33.9	3.0	66.4	N-I>S=Ave-A>N-C=P-A>P-L

P-A, Pembrolizumab-Axitinib; N-I, Nivolumab-Ipilimumab; Ave-A, Avelumab-Axitinib; N-C, Nivolumab-Cabozantinib; P-L, Pembrolizumab-Lenvatinib; S, Sunitinib.

a Preferred rank probabilities of interventions for study endpoints.

b SUCRA of interventions for study endpoints.

### Progression-free survival

**Figure 3:**
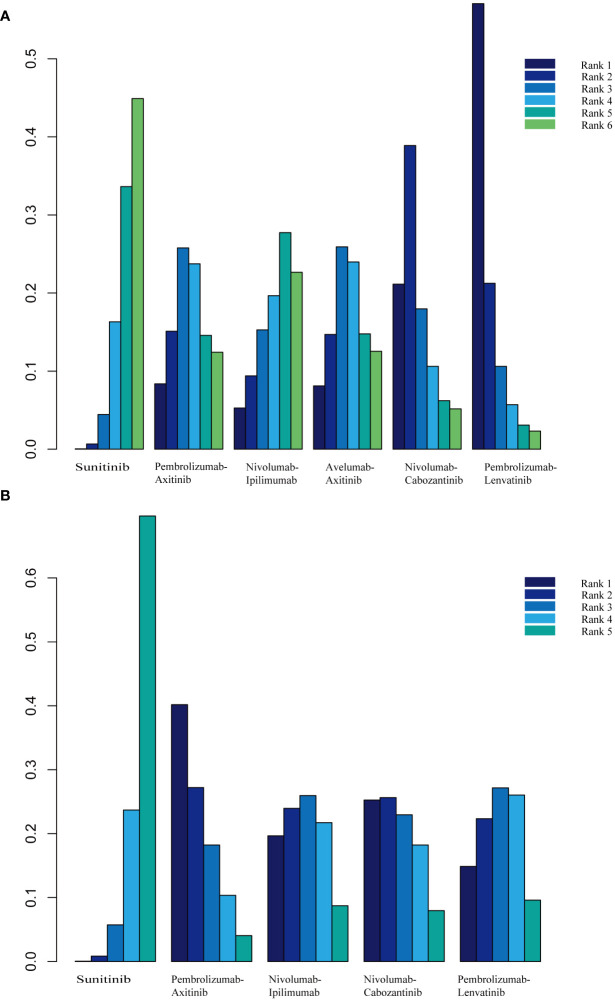
**(A)** Rank probabilities of the studied interventions for progression-free survival. **(B)** Rank probabilities of the studied interventions for overall survival.

PFS was the primary endpoint in all aRCC trials. Benefits of ICI-based combinations with respect to PFS were observed. In an indirect comparison, nivolumab plus cabozantinib and pembrolizumab plus lenvatinib appeared to be the most efficacious treatments; nivolumab plus cabozantinib showed an efficacy advantage in prolonging PFS in a point-by-point comparison with either pembrolizumab plus axitinib, nivolumab plus ipilimumab, or avelumab plus axitinib. PFS was also in favor of pembrolizumab plus lenvatinib compared to pembrolizumab plus axitinib, nivolumab plus ipilimumab, or avelumab plus axitinib (**Table 3**). However, no indirect comparative differences were observed between the nivolumab plus cabozantinib and pembrolizumab plus lenvatinib groups (HR, 1.31; 95% CI, 0.96–1.78). The rank probability analysis revealed a preference for pembrolizumab plus lenvatinib (57.1%) over nivolumab plus cabozantinib (21.1%). Moreover, pembrolizumab plus axitinib (8.4%), nivolumab plus ipilimumab (5.3%), and avelumab plus axitinib (8.1%) had similar selection probabilities (**Figure 3A**).

### Overall survival

The OS outcomes were better than those of sunitinib for each ICI combination therapy (**Table 2**). However, we did not observe any differences in the efficacy of pembrolizumab plus axitinib, nivolumab plus ipilimumab, avelumab plus axitinib, nivolumab plus cabozantinib, or pembrolizumab plus lenvatinib in the indirect comparisons (**Table 3**). Pembrolizumab plus axitinib (40.2%) was selected as the first treatment option following rank probability analysis, followed by nivolumab plus cabozantinib (25.3%), nivolumab plus ipilimumab (19.7%), and pembrolizumab plus lenvatinib (14.9%) (**Figure 3B**).

### Objective response rate

**Figure 4:**
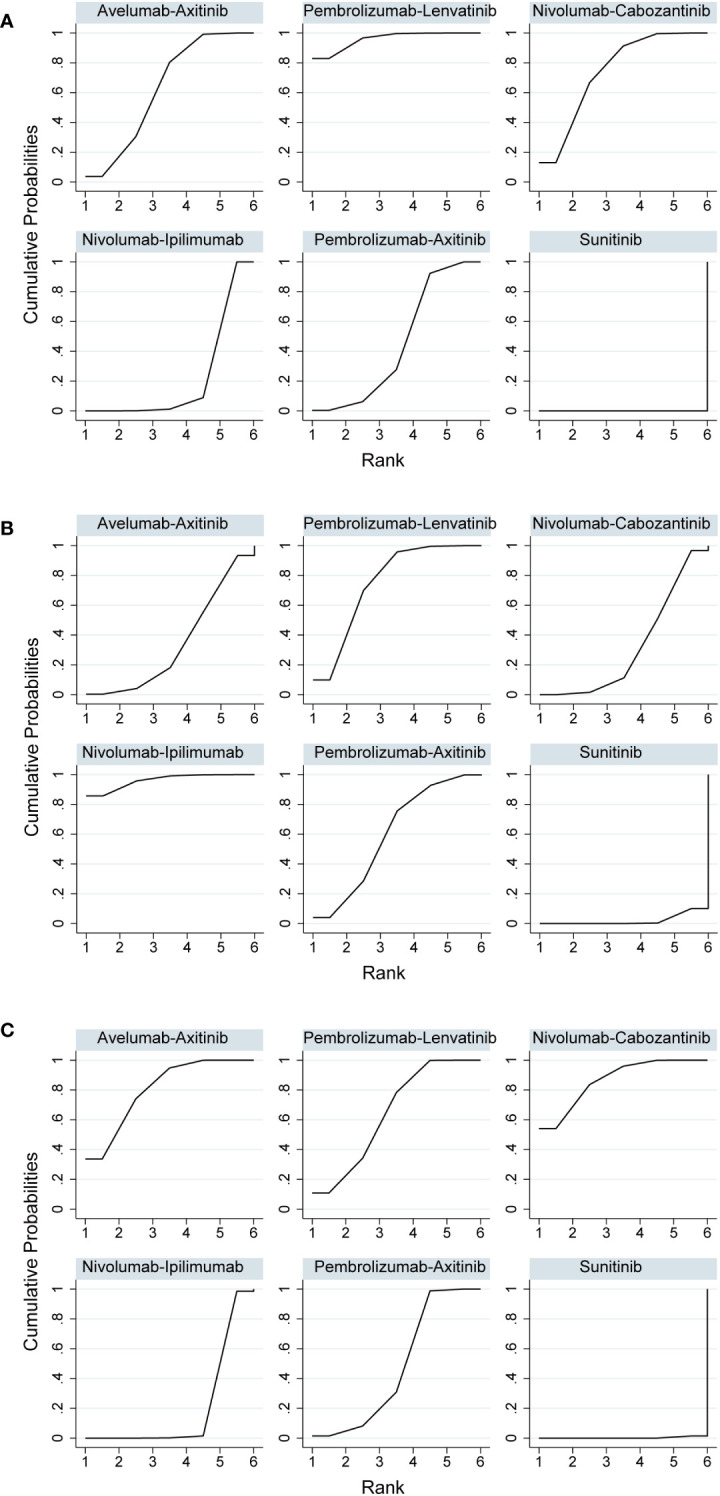
**(A)** Rank probabilities of the studied interventions for objective response rate. **(B)** Rank probabilities of the studied interventions for complete response. **(C)** Rank probabilities of the studied interventions for partial response.

The number of events in each arm of the included studies was used to calculate the OR and effect size for indirect comparisons. ORR was unfavorable to nivolumab plus ipilimumab compared to avelumab plus axitinib (OR, 0.64; 95% CI, 0.43–0.96), nivolumab plus cabozantinib (OR, 0.58; 95% CI, 0.38–0.90), or pembrolizumab plus lenvatinib (OR, 0.45; 95% CI, 0.30–0.70). However, treatment with avelumab plus axitinib, nivolumab plus cabozantinib, or pembrolizumab plus lenvatinib did not differ significantly between the groups (**Table 3**). Pembrolizumab plus lenvatinib showed the highest SUCRA value (95.8%), followed by nivolumab plus cabozantinib, avelumab plus axitinib, pembrolizumab plus axitinib, and nivolumab plus ipilimumab (**Table 4**; **Figure 4A**).

For indirect comparison of CR, only nivolumab plus ipilimumab versus nivolumab plus cabozantinib was statistically significant; nivolumab plus ipilimumab was better than nivolumab plus cabozantinib (OR, 4.74; 95% CI, 1.51–14.92). Nivolumab plus ipilimumab had the highest SUCRA of all interventions (96.1%) (**Figure 4B**). Similar to the ORR outcomes, pembrolizumab plus axitinib, avelumab plus axitinib, nivolumab plus cabozantinib, and pembrolizumab plus lenvatinib were preferable to nivolumab plus ipilimumab in partial response, and there was no significant difference between these four interventions (**Table 3**). Nivolumab plus cabozantinib and avelumab plus axitinib had a similar SUCRA value (86.7% and 80.5%) followed by pembrolizumab plus lenvatinib at 64.7% (**Figure 4C**).

### Adverse events

**Figure 5:**
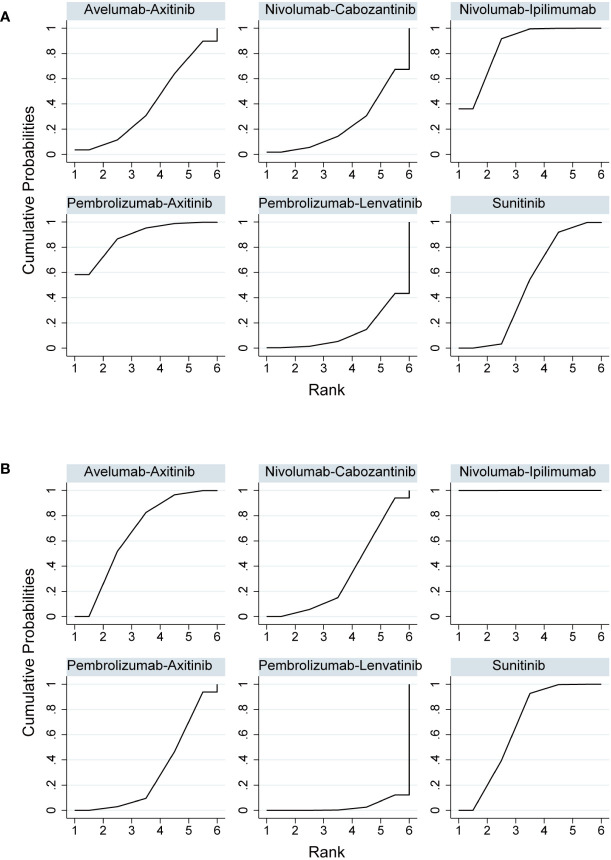
**(A)** Rank probabilities of the studied interventions for overall adverse events. **(B)** Rank probabilities of the studied interventions for adverse events ≥3.

In indirect comparisons, there was a lower incidence of overall AEs for pembrolizumab plus axitinib (OR, 0.05; 95% CI, 0.00–0.78) and nivolumab plus ipilimumab (OR, 0.07; 95% CI, 0.01–0.65) than pembrolizumab plus lenvatinib; however, there was no significant difference between pembrolizumab plus axitinib and nivolumab plus ipilimumab (**Table 3**). The SUCRA values for the probability of the lowest overall AE rates for pembrolizumab plus axitinib and nivolumab plus ipilimumab were 87.8% and 85.5%, respectively (**Table 4**; **Figure 5A**). Based on the comparison results, nivolumab plus ipilimumab had fewer AEs ≥3 than the other four interventions and sunitinib (**Figure 5B**). A SUCRA value of 100.0% also proved that nivolumab plus ipilimumab was the most preferred treatment. Additionally, the probability of AEs ≥3 were greater for pembrolizumab plus lenvatinib than for sunitinib (OR, 1.84; 95% CI, 1.28–2.64).

### Subgroup analysis

Age and sex were used in the subgroup analysis. Only pembrolizumab plus axitinib, avelumab plus axitinib, nivolumab plus cabozantinib, and pembrolizumab plus lenvatinib involved subgroup analysis of PFS. Consistent with previous analyses, nivolumab plus cabozantinib and pembrolizumab plus lenvatinib showed an efficacy advantage; PFS was in favor of nivolumab plus cabozantinib and pembrolizumab plus lenvatinib compared to pembrolizumab plus axitinib in patients aged < 65 years or in males (**Table 3**). In addition, pembrolizumab plus lenvatinib was better than avelumab plus axitinib in patients aged < 65 years and in both sexes. We could not find any differences among the four interventions in patients ≥ 65 years of age.

Pembrolizumab plus axitinib, nivolumab plus ipilimumab, and nivolumab plus cabozantinib were used for the subgroup analysis of OS. However, no significant differences were found among the three interventions in any subgroup (**Table 3**).

## Discussion

By 2005, evidence supported the idea that the induction of vascular normalization by anti-angiogenic medicines enhanced the effects of radiotherapy, immunotherapy, and chemotherapy (19, 20). Therefore, VEGF inhibitors and ICIs may work in combination to treat tumor microenvironments. Anti-VEGF TKIs may restore normal tissue permeability and vascularization, allowing an inflow of immune cells into the tumor stroma, whereas ICIs may revive the immune system in tumor microenvironments (21, 22). After a decade of sequential monotherapy, combination therapies are now the standard of care for all first-line treatment of patients with metastatic clear-cell renal cell carcinoma (mccRCC). ICI-TKI combinations are indicated for all patients, and ICI-ICI combinations are only indicated for patients with intermediate and poor prognosis (23). However, there are wide variations in the markers measured, assays used, and evaluation of tumors and/or immune cells; thus, determining the optimal drug combination is imperative (24). In this study, PFS and OS were the primary endpoints. Initial data reported that patients with aRCC who received first-line combination therapies had significantly longer PFS than those who received sunitinib. Our network meta-analysis demonstrated that nivolumab plus cabozantinib or pembrolizumab plus lenvatinib resulted in a better PFS. However, because of the lack of statistically significant indirect comparative differences between nivolumab plus cabozantinib and pembrolizumab plus lenvatinib, we concluded by rank probability analysis that pembrolizumab plus lenvatinib (57.1%) was the optimal choice in clinical setting, followed by nivolumab plus cabozantinib. Further, the results of the median PFS for all reported trials were comparable. In the CLEAR study, PFS for pembrolizumab plus lenvatinib was 23.9 months, while the CheckMate 9ER study reported 16.6 months for nivolumab plus cabozantinib. Pembrolizumab plus axitinib, nivolumab plus ipilimumab, and avelumab plus axitinib were not significantly different in indirect comparisons, and the rank probabilities were similar. Therefore, more endpoints need to be analyzed to determine the difference between them.

OS was also better with the combination treatment. Subgroup analysis supported the benefit of nivolumab plus cabozantinib over sunitinib in terms of OS, regardless of key baseline characteristics. These results suggest that cabozantinib may enhance ICIs and that the synergistic effects of TKIs may increase the efficacy of ICIs (25). However, the analysis of OS in preliminary data was immature. None of the intervention groups in the study reached the median OS. Only patients in the CheckMate 214 trial who received sunitinib had a median OS of 26.0 months. We did not find any differences in OS by indirect comparison of the interventions. The rank probabilities suggested that pembrolizumab plus axitinib (40.2%) might be a better treatment, although the findings need to be viewed with caution.

The use of subsequent lines of therapy, patient crossover and/or patient access to the investigational medication for patients in the control arms, difficulties with patient follow-up, and greater post-progression survival can frequently skew OS data (26). Given that ORR and PFS allow for shorter trial durations and the use of smaller patient cohorts, particularly in the case of ORR, potentially allowing for single-arm trial designs, and an urgent need for new cancer therapies, the FDA has expedited approval by allowing the use of ORR and PFS endpoints as surrogates for OS (27). In all included studies, the ORR was higher in the combination treatment group. Our analysis showed that pembrolizumab plus lenvatinib for ORR and nivolumab plus cabozantinib for partial response had the highest probability of being the best treatment strategies, and nivolumab plus ipilimumab had the lowest ranking in the treatment selection priority order for ORR and partial response. However, this result was reversed for CR, with nivolumab plus ipilimumab emerging as the best choice. For nivolumab plus ipilimumab, as the only ICI-ICI combination in the study, we only studied the IMDC defined intermediate- and poor-risk patients (CheckMate 214). Contrarily, the favorable-risk group had a higher ORR and longer PFS with sunitinib than with nivolumab plus ipilimumab (29.0% vs. 52.0%), which requires further interpretation because of insufficient survival data and a small sample size. This difference also suggests the need to elucidate the underlying biological processes of nivolumab plus ipilimumab. A recent study showed that significant gains in the ORR were indicative of possible OS improvement, with a negligible ORR benefit not ruling out an OS benefit (28). Given the high objective response rate in the combination therapy group, it seems feasible that combination therapy with ICIs could lead to an OS benefit.

The benefits of combination therapy must be individually weighed against the frequency and severity of AEs. PD-1 blocks T cells at a later stage of the immune cascade response in peripheral tissues and CTLA-4 attenuates T cell activation at the proximal step of the immune response (29), the differences of which are reflected in the AEs. For example, anti-PD-1 therapy appears to increase the prevalence of pneumonitis and thyroiditis, whereas anti-CTLA-4 therapy appears to increase the prevalence of colitis and hypophysitis (30). The incidence of AEs observed with ICI-based combinations was generally consistent with the known safety profile of an ICI and a TKI as monotherapy or in combination (31); the most common AEs involved the skin (rash, pruritus), gastrointestinal tract (colitis, diarrhea), liver (hepatitis), endocrine system (thyroid disease), and lungs (pneumonia). The overall incidence of AEs for the ICI-TKI combination was 95–97% for all grades and 57–72% for grade ≥3, and 94% for all grades of toxicity with the ICI-ICI combination. Our study showed the lowest incidence of overall AEs with pembrolizumab plus axitinib, whereas nivolumab plus ipilimumab had a definite selective advantage with a lower incidence of AEs ≥3. Drug combinations with different grades of AEs suggests that attention should be paid to the overlapping toxicity profiles of ICIs and TKIs in combination therapy for aRCC. In clinical settings, TKI therapy should be discontinued, and ICI infusion delayed if it is not determined whether the AEs were caused by either of these agents; AEs caused by a TKI are expected to improve. In addition, the type and severity of AEs and the general condition of the patient must be considered in view of the initiating corticosteroid therapy to prevent worsening of potential toxicity. If symptoms subside rapidly after corticosteroid treatment, the AEs will have most likely been immune-related (32).

Although ICIs have enabled some patients to achieve previously unattainable levels of survival, the emergence of drug resistance over time cannot be ignored. The response rates for ICIs-treated aRCC are 5-27%, meaning that only a small percentage of people actually achieve a durable response (33). Resistance to ICIs therapy has been classified into primary resistance and acquired resistance (34). Primary resistance is characterized by an immediate lack of response to therapeutic compounds when tumor cells do not express the intended target or are intrinsically resistant cells. By contrast, acquired resistance occurs while the patient is still receiving treatment during the course of the disease and is characterized by disease progression and cancer recurrence after initial tumor regression. Mechanisms by which tumors evade ICIs leading to drug resistance include lack of T cell priming and impaired antigen presentation; reduced T cell activity or absence of T cells in the tumor microenvironment (35). And most current guidelines recommend clinical trial participation or single-agent TKIs (with or without mTOR inhibition where indicated) as a subsequent therapy when disease progression occurs in patients treated with ICI-based combinations therapy (36, 37). Recently developed approaches to overcome ICIs resistance have focused on the tumor microenvironment. For example, expression of colony-stimulating factor 1 receptor allows for the conversion of type I macrophages to type II tumor-associated macrophages, which in turn promotes tumor neovascularization and progression (38). Also, Belzutifan inhibits hypoxia-inducible factor-2α by impairing the hypoxia signaling pathway in cancer cells, resulting in antitumor activity (39). The potential role of the gut microbiome in modulating ICI resistance in RCC is another concern. Recent studies show that increased gut microbial diversity is associated with better response to ICIs (40). In a randomized phase 1 trial, metastatic RCC patients who received nivolumab-ipilimumab with CBM 588 (a bifidogenic live bacterial product) had a higher response rate (58% vs. 20%, P = 0.06) compared to those receiving nivolumab-ipilimumab only (41). Despite the comprehensive nature of this systematic review, some limitations need to be considered. First, the follow-up durations of the included trials were relatively short. The median overall survival was not reached in either group, making the results of our indirect comparison of OS immature and requiring the assessment of tumor response to determine long-term outcomes. Second, we did not perform subgroup analysis based on the prognostic risk of the IMDC. To date, few studies have specifically addressed the efficacy of ICIs as treatments for intermediate- and poor-risk patients. Herein, data were also extracted from only intermediate- and poor-risk patients in the nivolumab plus ipilimumab study. The depth and durability of the responses reported for patients with different risk scores appeared to be different; therefore, treatment options for patients with different risk scores should be explored further. Third, the indirect comparative analyses were far from head-to-head treatment comparisons, and well-designed comparative trials are required to validate the results of this study.

Despite these limitations, we carefully selected the evidence, and the studies screened were high-quality randomized controlled trials with similar patient selection criteria. We not only demonstrated the utility of the combination of ICIs in extending survival in patients with aRCC, but also provide a basis for clinicians to study specific endpoints. Future research should concentrate on developing novel and more effective ways to manipulate the immune system for therapeutic purposes as well as ways to better stratify patients so that these therapies can be chosen and given priority for those who will benefit from them the most.

## Conclusion

Our network meta-analysis revealed that combination therapy with an ICI prolongs PFS and OS in patients with aRCC. Based on indirect comparisons and probability analyses, the most effective treatment options are pembrolizumab plus lenvatinib for PFS, and pembrolizumab plus axitinib for OS. Nonetheless, toxicity also needs to be considered; the use of pembrolizumab plus axitinib causes fewer overall AEs, whereas nivolumab plus ipilimumab is the best option to avoid tertiary AEs ≥3.

## Data availability statement

The original contributions presented in the study are included in the article/**Supplementary Material**. Further inquiries can be directed to the corresponding author.

## Author contributions

XC: Conceptualization, Data curation, Formal analysis, Investigation, Methodology, Project administration, Software, Supervision, Validation, Writing – original draft, Writing – review & editing. ZX: Resources, Software, Validation, Writing – original draft, Writing – review & editing. CW: Investigation, Validation, Writing – original draft, Writing – review & editing. LX: Resources, Writing – review & editing. PW: Validation, Writing – review & editing. XL: Writing – original draft, Writing – review & editing.
