# Supplementary material for: Efficacy and toxicity of immune checkpoint inhibitors combination therapy for advanced renal cell carcinoma: a systematic review and network meta-analysis
Source: Front Immunol. 2024 Feb 8;15:1255577. doi: 10.3389/fimmu.2024.1255577 (PMC10881808; doi:10.3389/fimmu.2024.1255577)
Supplement: Supplementary file 1 [file DataSheet_1.docx]

***Supplementary Material***

**Search strategy**

**Pubmed**

#1 “Immune Checkpoint Inhibitors”[MeSH Terms] OR Immune Checkpoint Inhibitor[All Fields] OR Immune Checkpoint Blockers[All Fields] OR Immune Checkpoint Blockade[All Fields] OR Immune Checkpoint Inhibition[All Fields] OR PD-L1 Inhibitors[All Fields] OR Programmed Death-Ligand 1 Inhibitors[All Fields] OR PD-1-PD-L1 Blockade[All Fields] OR CTLA-4 Inhibitors[All Fields] OR Cytotoxic T-Lymphocyte-Associated Protein 4 Inhibitors[All Fields] OR PD-1 Inhibitors[All Fields] OR Programmed Cell Death Protein 1 Inhibitors[All Fields]

#2 “Sunitinib”[MeSH Terms] OR 5-(5-Fluoro-2-oxo-1,2-dihydroindolylidenemethyl)-2,4-dimethyl-1H-pyrrole-3-carboxylic acid (2-diethylaminoethyl)amide[All Fields] OR Sunitinib Malate[All Fields] OR Sutent[All Fields] OR SU 11248[All Fields] OR SU011248[All Fields] OR SU-011248[All Fields]

#3 “Pembrolizumab”[MeSH Terms] OR SCH-900475[All Fields] OR lambrolizumab[All Fields] OR MK-3475[All Fields] OR Keytruda[All Fields]

#4 “Nivolumab”[MeSH Terms] OR Opdivo[All Fields] OR ONO-4538[All Fields] OR MDX-1106[All Fields] OR BMS-936558[All Fields]

#5 “avelumab”[MeSH Terms] OR MSB-0010682[All Fields] OR bavencio[All Fields] OR MSB0010718C[All Fields]

#6 “Ipilimumab”[MeSH Terms] OR Anti-CTLA-4 MAb Ipilimumab[All Fields] OR Anti CTLA 4 MAb Ipilimumab[All Fields] OR Yervoy[All Fields] OR MDX 010[All Fields] OR MDX-010[All Fields] OR MDX-CTLA-4[All Fields] OR MDX CTLA 4[All Fields]

#7 “Carcinoma, Renal Cell”[MeSH Terms] OR Renal Cell Carcinomas[All Fields] OR Nephroid Carcinoma[All Fields] OR Adenocarcinoma Of Kidney[All Fields] OR Renal Cell Cancer[All Fields] OR Renal Adenocarcinoma[All Fields] OR Renal Carcinoma[All Fields] OR Renal Cell Adenocarcinoma[All Fields] OR Chromophobe Renal Cell Carcinoma[All Fields] OR Sarcomatoid Renal Cell Carcinoma[All Fields] OR Papillary Renal Cell Carcinoma[All Fields] OR Chromophil Renal Cell Carcinoma[All Fields] OR Clear Cell Renal Cell Carcinoma[All Fields] OR Grawitz Tumor[All Fields] OR Clear Cell Renal Carcinoma[All Fields] OR Hypernephroid Carcinoma[All Fields] OR Hypernephroma[All Fields] OR Collecting Duct Carcinoma (Kidney)[All Fields] OR Collecting Duct Carcinoma of the Kidney[All Fields] OR Renal Collecting Duct Carcinoma[All Fields] OR Collecting Duct Carcinoma[All Fields]

#8 ((randomized controlled trial[pt] OR controlled clinical trial[pt] OR randomized[tiab] OR randomised[tiab] OR placebo[tiab] OR drug therapy[sh] OR randomly[tiab] OR trial[tiab] OR groups[tiab]) NOT (animals[mh] NOT humans[mh]))

#9 #1 OR #2 OR #3 OR #4 OR #5 OR #6

#10 #7 AND #8 AND #9

**EMBASE**

1 Randomized controlled trial/

2 Controlled clinical study/

3 Random$.ti,ab.

4 randomization/

5 intermethod comparison/

6 placebo.ti,ab.

7 (compare or compared or comparison).ti.

8 (open adj label).ti,ab.

9 ((double or single or doubly or singly) adj (blind or blinded or blindly)).ti,ab.

10 double blind procedure/

11 parallel group$1.ti,ab.

12 crossover or cross over).ti,ab.

13 ((assign$ or match or matched or allocation) adj5 (alternate or group$1 or intervention$1 or patient$1 or subject$1 or participant$1)).ti,ab.

14 (assigned or allocated).ti,ab.

15 (controlled adj7 (study or design or trial)).ti,ab.

16 (volunteer or volunteers).ti,ab.

17 trial.ti.

18 or/1-17

19 exp renal/

20 exp renal disease/

21 (19 or 20) and exp neoplasm/

22 exp renal cell tumor/

23 exp renal cell cancer/

24 exp renal cell carcinoma/

25 (renal cell$ adj5 (neoplas$ or cancer$ or carcin$ or tumo$ or metasta$ or malig$)).ti,ab.

26 (advanced adj5 (renal cell adj5 (neoplasm$ or cancer$ or carcinoma$ or tumo?r$))).ti,ab.

27 or/21-26

28 exp Programmed Death-Ligand 1 Inhibitors/

29 exp Cytotoxic T-Lymphocyte-Associated Protein 4 Inhibitors/

30 exp Programmed Cell Death Protein 1 Inhibitor/

31 immune checkpoint inhibitor$.mp.

32 (immune adj5 checkpoint adj5 inhibitor$).mp.

33 exp pembrolizumab /

34 (pembrolizumab or SCH-900475 or MK-3475 or lambrolizumab or keytruda).mp.

35 exp nivolumab /

36 (nivolumab or Opdivo or ONO-4538 or MDX-1106 or BMS-936558).mp.

37 exp avelumab/

38 (avelumab or MSB-0010682 or bavencio or MSB0010718C).mp.

39 exp ipilimumab /

40 (ipilimumab or Anti-CTLA-4 MAb Ipilimumab or Yervoy or MDX 010 or MDX-CTLA-4).mp.

41 or/28-40

42 18 and 27 and 41

43 limit 42 to (human and (conference abstracts or embase))

44 limit 43 to yr=”2020-Current”

**The Cochrane Library**

#1 MeSH descriptor: [Carcinoma, Renal Cell] explode all trees

#2 metasta* near renal cell near cancer* or metastatic renal cell cancer*

#3 metasta* near renal cell near neoplasm* or metastatic renal cell neoplasm*

#4 metasta* near renal cell near carcinom* or metastatic renal cell carcinom*

#5 metasta* near renal cell near tumour* or metastatic renal cell tumour*

#6 metasta* near renal cell near tumor* or metastatic renal cell tumor*

#7 #1 or #2 or #3 or #4 or #5 or #6

#8 MeSH descriptor: [Immune Checkpoint Inhibitor] explode all trees

#9 ‘Programmed Death-Ligand 1 Inhibitor’ or Programmed Death-Ligand 1* near inhibitor*

#10 ‘Cytotoxic T-Lymphocyte-Associated Protein 4 Inhibitor’ or Cytotoxic T-Lymphocyte-Associated Protein 4* near inhibitor*

#11 ‘Programmed Cell Death Protein 1 Inhibitor’ or Programmed Cell Death Protein 1* near inhibitor*

#12 PD-L1* inhibitor* or PD-L1* near inhibitor*

#13 CTLA-4* inhibitor* or CTLA-4* near inhibitor*

#14 PD-1* inhibitor* or PD-1* near inhibitor*

#15 pembrolizumab or SCH-900475 or MK-3475

#16 nivolumab or ONO-4538 or MDX-1106 or BMS-936558

#17 avelumab or MSB-0010682 or MSB0010718C

#18 ipilimumab or Anti-CTLA-4 MAb Ipilimumab or MDX 010 or MDX-CTLA-4

#19 #8 or #9 or #10 or #11 #12 or #13 or #14 or #15 or #16 or #17 or #18

#20 #7 and #19
